# Supplementary material for: Association between oral microbiome and seven types of cancers in East Asian population: a two-sample Mendelian randomization analysis
Source: Front Mol Biosci. 2023 Nov 21;10:1327893. doi: 10.3389/fmolb.2023.1327893 (PMC10702768; doi:10.3389/fmolb.2023.1327893)
Supplement: Supplementary file 1 [file DataSheet1.ZIP › Supplementary Materials/MR plots of saliva/saliva═╝/ovarian cancer/pheno.743_to_ovarian cancer_leave_one_out.pdf]

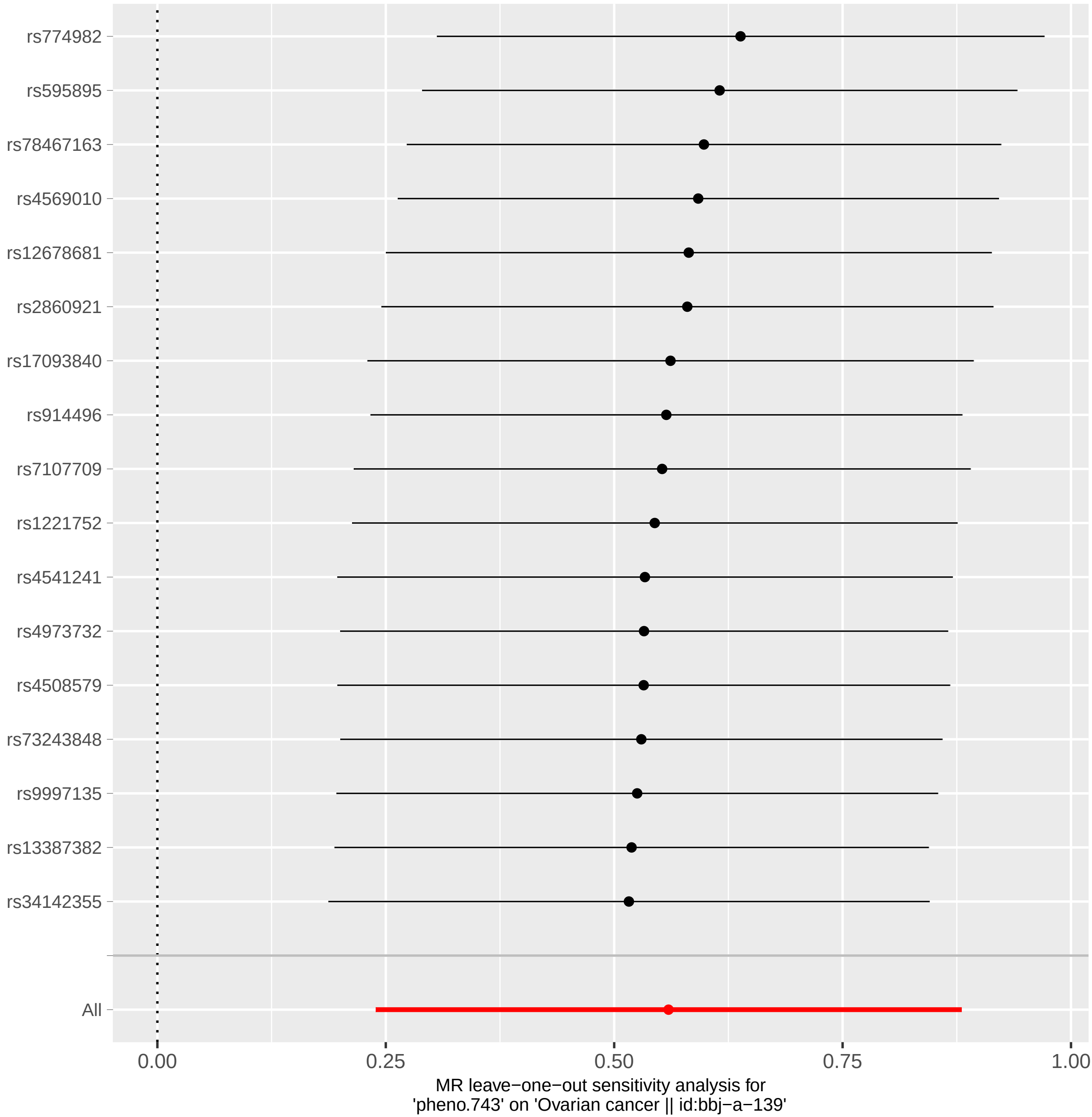
rs774982
rs595895
rs78467163
rs4569010
rs12678681
rs2860921
rs17093840
rs914496
rs7107709
rs1221752
rs4541241
rs4973732
rs4508579
rs73243848
rs9997135
rs13387382
rs34142355
All
0.00
0.25
0.50
0.75
1.00
MR leave−one−out sensitivity analysis for
'pheno.743' on 'Ovarian cancer || id:bbj−a−139'
